# Recognition of Intensive Valence and Arousal Affective States via Facial Electromyographic Activity in Young and Senior Adults

**DOI:** 10.1371/journal.pone.0146691

**Published:** 2016-01-13

**Authors:** Jun-Wen Tan, Adriano O. Andrade, Hang Li, Steffen Walter, David Hrabal, Stefanie Rukavina, Kerstin Limbrecht-Ecklundt, Holger Hoffman, Harald C. Traue

**Affiliations:** 1 College of Teacher Education, Lishui University, Lishui, P.R. China; 2 Biomedical Engineering Laboratory, Faculty of Electrical Engineering, Federal University of Uberlândia, Uberlândia, Brazil; 3 Emotion Lab, Section Medical Psychology, University of Ulm, Ulm, Germany; Aristotle University of Thessaloniki, GREECE

## Abstract

**Background:**

Research suggests that interaction between humans and digital environments characterizes a form of companionship in addition to technical convenience. To this effect, humans have attempted to design computer systems able to demonstrably empathize with the human affective experience. Facial electromyography (EMG) is one such technique enabling machines to access to human affective states. Numerous studies have investigated the effects of valence emotions on facial EMG activity captured over the corrugator supercilii (frowning muscle) and zygomaticus major (smiling muscle). The arousal emotion, specifically, has not received much research attention, however. In the present study, we sought to identify intensive valence and arousal affective states via facial EMG activity.

**Methods:**

Ten blocks of affective pictures were separated into five categories: neutral valence/low arousal (0VLA), positive valence/high arousal (PVHA), negative valence/high arousal (NVHA), positive valence/low arousal (PVLA), and negative valence/low arousal (NVLA), and the ability of each to elicit corresponding valence and arousal affective states was investigated at length. One hundred and thirteen participants were subjected to these stimuli and provided facial EMG. A set of 16 features based on the amplitude, frequency, predictability, and variability of signals was defined and classified using a support vector machine (SVM).

**Results:**

We observed highly accurate classification rates based on the combined corrugator and zygomaticus EMG, ranging from 75.69% to 100.00% for the baseline and five affective states (0VLA, PVHA, PVLA, NVHA, and NVLA) in all individuals. There were significant differences in classification rate accuracy between senior and young adults, but there was no significant difference between female and male participants.

**Conclusion:**

Our research provides robust evidences for recognition of intensive valence and arousal affective states in young and senior adults. These findings contribute to the successful future application of facial EMG for identifying user affective states in human machine interaction (HMI) or companion robotic systems (CRS).

## Introduction

Alongside the rapid and extensive development of interactive devices such as tablets and smartphones, the interaction between humans and their digital environment has become not only a technical activity, but also a form of empathic companionship; the literature characterizes this relationship as human computer interaction (HCI) or generalized human machine interaction (HMI). This type of relationship provides not only passive functionalities, but also, ideally, functions based on perception of the user’s implicit current needs, responses, preferences, coherence, and intention [1–2]. This type of companionship-by-design system should be able to completely and individually adapt to the user, and reflect the user’s situation and emotional states; in other words, the system should have the ability to be empathetic [3–5].

To achieve an ideal technical companionship system, the user’s affective experience is a vital consideration; accurate and appropriate recognition of the user’s emotional states is paramount to the success of this type of technology. In human-human interaction (HHI), individuals recognize emotional states using innate sensors for facial expression, gestures, eye contact, language, speed or tone of speaking, and other natural indicators[6]. A computer, of course, is unable to perceive emotional states by definition. There are numerous signals relevant to emotional responses that are, though, measurable by devices such as cameras, microphones, and sensors [7]. For example, small electrodes attached to the skin can accurately detect psychobiological changes indicative of the human emotional experience, gaining access to human emotional states. One such psychophysiological sensor, the facial EMG, represents a robust and effective method of recognizing human affective states in HMI [8–9].

Affective responses involve subjective experience, central and peripheral nervous system changes, and behaviors (e.g., facial expressions, gestures, and vocal characteristics) [10]. Facial expressions, specifically, are innate and untrained reactions to affective states [11] which enable us to recognize and communicate emotions transiently as we interact with other people. Affective science research has struggled to quantifiably measure the affective states of human beings, however [10]. There are two primary issues related to this problem (and, as such, to the present study’s primary objectives). First, there is no standardized model for evaluating or interpreting emotions or affects [12–13], however, as Mauss and Robinson [10] suggested, “measures of affective responses seem to be structured along dimensions rather than discrete emotions”. Second, due to the sizeable (and rapidly growing) proportion of senior adults in many societies, particularly in Western countries, companion technology is highly desirable because of a shortage of qualified healthcare personnel [9]. A meta-analytic study reviewing emotions and aging suggested that seniors are worse than younger adults in recognizing emotional states, and that there is a general declining trend in emotion recognition with age [14], though the results of this study may have not been fully valid. Aging does indeed play an essential role in detecting affective responses, however.

The tridimensional theory of emotion, which is commonly applied in studies on this subject, evaluates affective states according to valence, arousal, and dominance (VAD). This approach can be dated back to Wundt [15]. “Valence” describes affective states from highly negative (unpleasant) to highly positive (pleasant); “arousal” measures the intensity of affective states ranging from highly calm to highly excited or alert; and “dominance” represents the feeling of being controlled or influenced by external stimuli [16–17]. Lang, Rice, and Sternbach assumed that emotion is comprised of these three dimensions [18]; and research has shown that dominance is highly correlated with the dimension of valence [16]. As the proponents of this theory have suggested, the tridimensional model of emotion can be reduced to two orthogonal dimensions [19–21]: valence and arousal, in which all emotions can be classified. We adopted the two-dimensional theory of affective experience in this study.

A number of researchers have investigated the effects of the sole dimension of valence on facial EMG. Their findings demonstrated that facial EMG captured over corrugator supercilii (frowning muscle), which is associated with negative emotional expressions, and zygomaticus major (smiling muscle), which is related to positive emotional expressions [22–23], can differentiate valence emotions and their intensities [24]. In these studies, corrugator EMG amplitude increased in response to negative affective stimuli and decreased with positive affective stimuli compared to neutral stimuli, whereas zygomaticus EMG amplitude increased during positive stimuli [25–29]. In addition, zygomaticus EMG amplitude was not shown to discriminate neutral and negative emotions, thus, corrugator and zygomaticus EMG activities can be considered indicators of negative and positive affective states, respectively [25,28].

To date, the dimension of arousal has not received much attention in facial EMG emotion recognition studies. Very limited evidence from previous studies has shown, though, that zygomaticus EMG differentiates positive valence/low arousal affective states from positive valence/high arousal affective conditions. In the participants in these studies, the zygomaticus EMG activity was lower when viewing positive valence/low arousal affective images than when viewing positive valence/high arousal ones [30], i.e., the high-arousal affective pictures elicited higher corrugator and zygomaticus EMG activities than low-arousal ones [31]. Corrugator and zygomaticus EMG could not independently differentiate the affective states in the arousal dimension [28], which may modulate facial EMG responses when participants are confronted by visual stimuli. Therefore, taking both the dimensions of valence and arousal simultaneously into account is arguably necessary for detecting their compound effects on facial EMG technology.

Research has also shown that increasing age is correlated with a general decline in physiological functions [32–33], and many previous studies have indicated lower physiological responses in senior adults than in young adults when exposed to affective stimuli [34]. Along with the decline in physiological functions, increased positive well-being, affect control, and affect regulation have been observed in senior compared to young adults [35–37]. Influenced by the “positivity effect”, seniors typically highly favor positive stimuli, whereas negative stimuli are more recognized by young adults [38]. Conversely, senior adults in certain situations experience greater affective responses than young adults do, although evidence suggests less affective reactions for senior adults than young adults in most contexts [39]. Researchers have claimed that there are no significant age variations in either corrugator or zygomaticus EMG in relation to valence affective states [40], but researchers have identified lower overall corrugator EMG activity in response to affective pictures in senior adults, regardless of valence [41]. Another study found that increasing age is associated with decreased corrugator EMG amplitude in response to neutral stimuli [42]. Evidence for age differences in facial EMG on intensive valence and arousal affective states, however, is scarce in the literature.

In brief, the primary goal of the present study was to assess the potentially distinct effects of intensive valence and arousal affective states on facial EMG detected over the corrugator supercilii and zygomaticus major of study participants. The secondary objective was to investigate age differences in the EMG activity between senior and young adults in different affective states, and the same by gender. We employed a case study followed by classification methods and statistical analysis to achieve these goals.

## Materials and Methods

### Participants

Seventy young adults (from 20 to 40 years of age, mean = 24.57, SD = 4.37) and 43 senior adults (from 52 to 77 years of age, mean = 64.30, SD = 7.16) participated in this study. The data from two subjects in the young group and three in the senior group were excluded from subsequent analysis due to technical problems (i.e., movements or artifacts.) The final sample size was 108 (69 females, 39 males). All participants were healthy and had normal vision or corrected normal vision. The experiment was designed and implemented according to the ethical guidelines of the University of Ulm and was approved by the university’s Ethical Committee (number: 245/08-UBB/se).

### Stimuli

**Fig 1:**
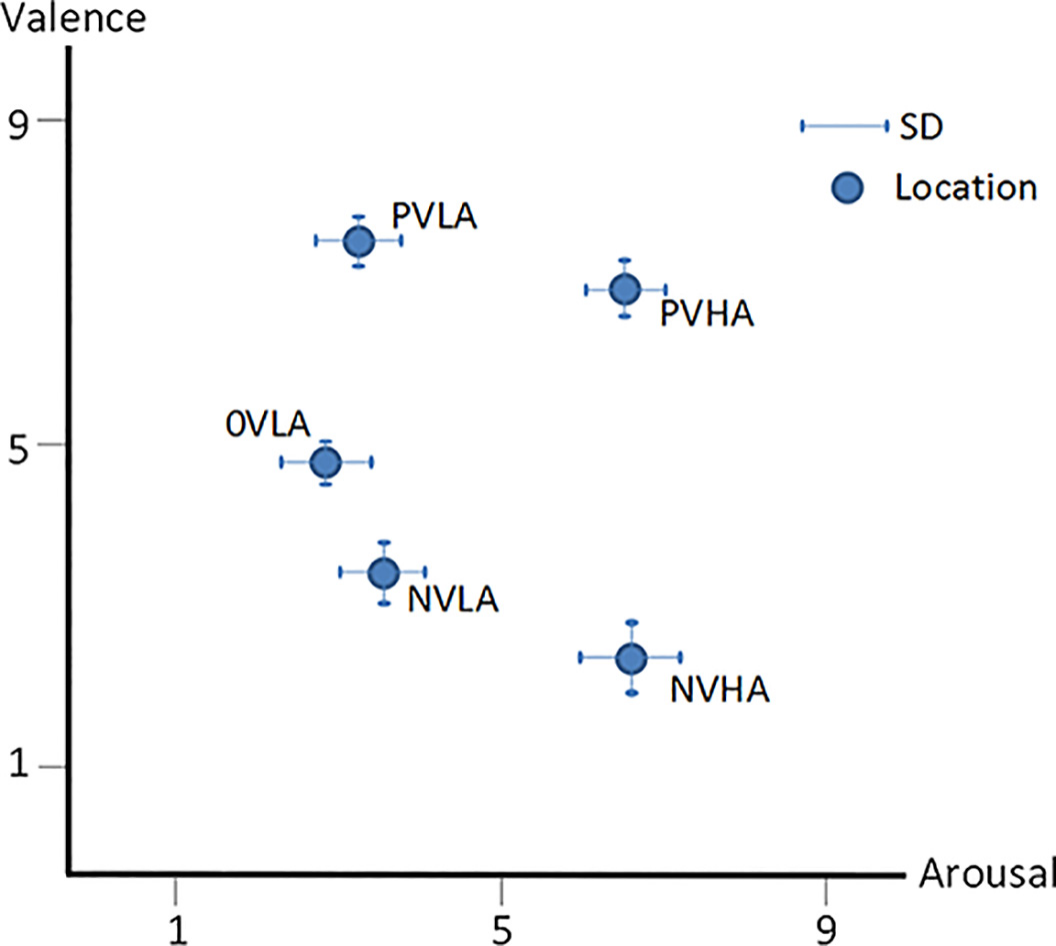
Location of affective stimuli in the two-dimensional space.

To elicit intensive and sustained affective states, which are not easily induced by a static single image in laboratory situations, the stimuli consisted of 10 picture blocks. In each block, 10 affective images with similar rating scores on valence and arousal were combined. Thus, a total of 100 pictures chosen from the international affective picture system (IAPS) [43] and Ulm pictures [44], which are well-designed, standardized, and generally employed for eliciting emotional states with three dimensions (VAD) and are often utilized in studies on affective science or affective computing. The content of the pictures ranged from daily experiences (e.g., household furniture) intended to elicit neutral and calm emotions, to extreme encounters (e.g., severe human injuries,) that induce highly negative and arousal emotions, or to erotic images intended to produce highly positive and arousal emotions. Ten picture blocks were divided into five categories (i.e., two picture blocks with similar valence-arousal responded to one of five affective states) according to the standardized rating scores: 1) 0VLA, neutral valence (4.96 ± 0.25) and low arousal (2.75 ± 0.64); 2) NVLA, negative valence (3.74 ± 0.43) and low arousal (3.63 ± 0.51); 3) NVHA, negative valence (2.20 ± 0.64) and high arousal (6.66 ± 0.57); 4) PVLA, positive valence (7.58 ± 0.39) and low arousal (3.26 ± 0.47); and 5) PVHA, positive valence (7.00 ± 0.57) and high arousal (6.50 ± 0.51). Fig 1 shows the location of these five categories of affective states in the valence-arousal space.

### Procedure

Participants were seated in a comfortable reclining chair in a sound-attenuated room of the Emotion Lab, Ulm, after being introduced to the experiment and signing an informed consent form. EMG sensors were attached to the participants’ respective facial muscles, afterwards participants were asked to relax, keep stable, and pay attention to the image blocks for the duration of the experiment.

**Fig 2:**
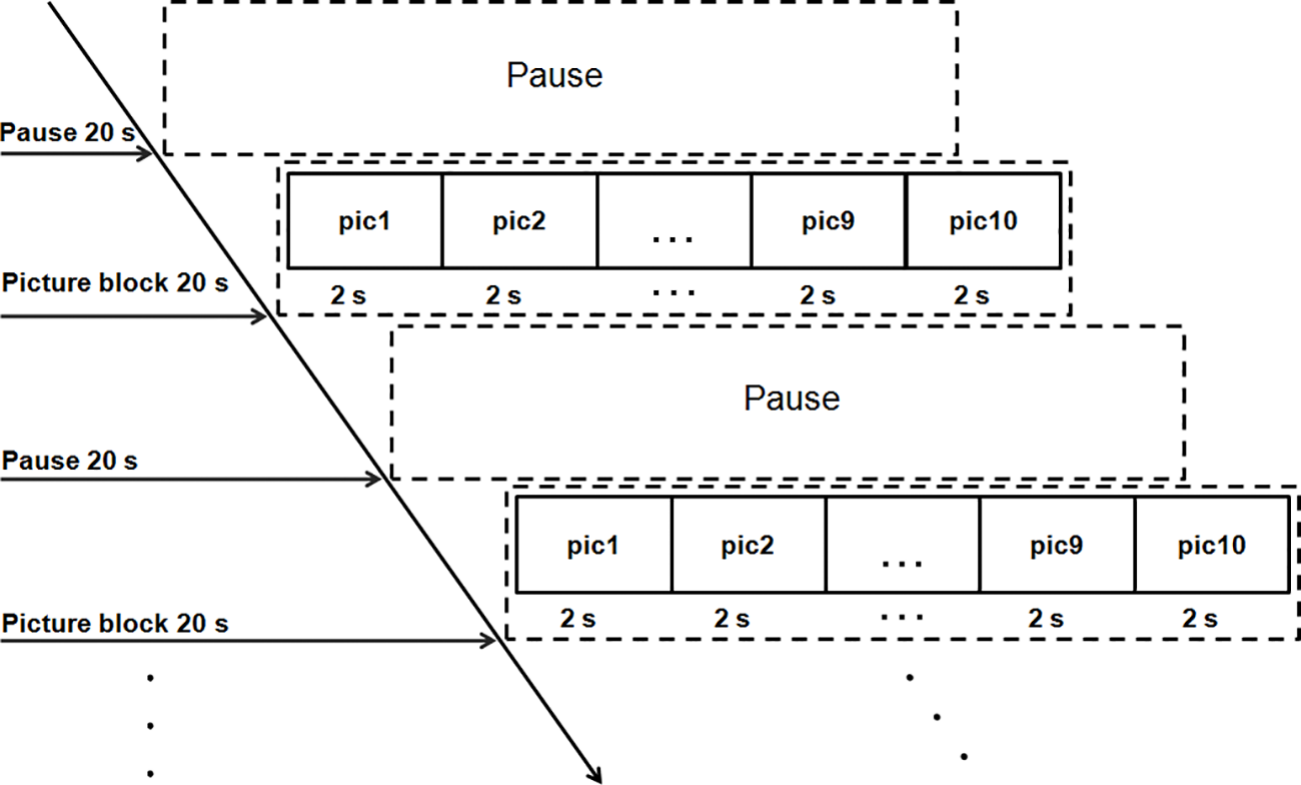
Presentation of picture blocks.

Ten affective image blocks were presented randomly on a 17-inch monitor. Each block, carrying the same probability without any repetition, was presented for 20 s, during which each of the 10 pictures was shown for 2 s continuously. There was a fixed pause of 20 s between blocks (Fig 2). Facial EMG activity was recorded throughout the experiment.

### EMG Data Acquisition

**Fig 3:**
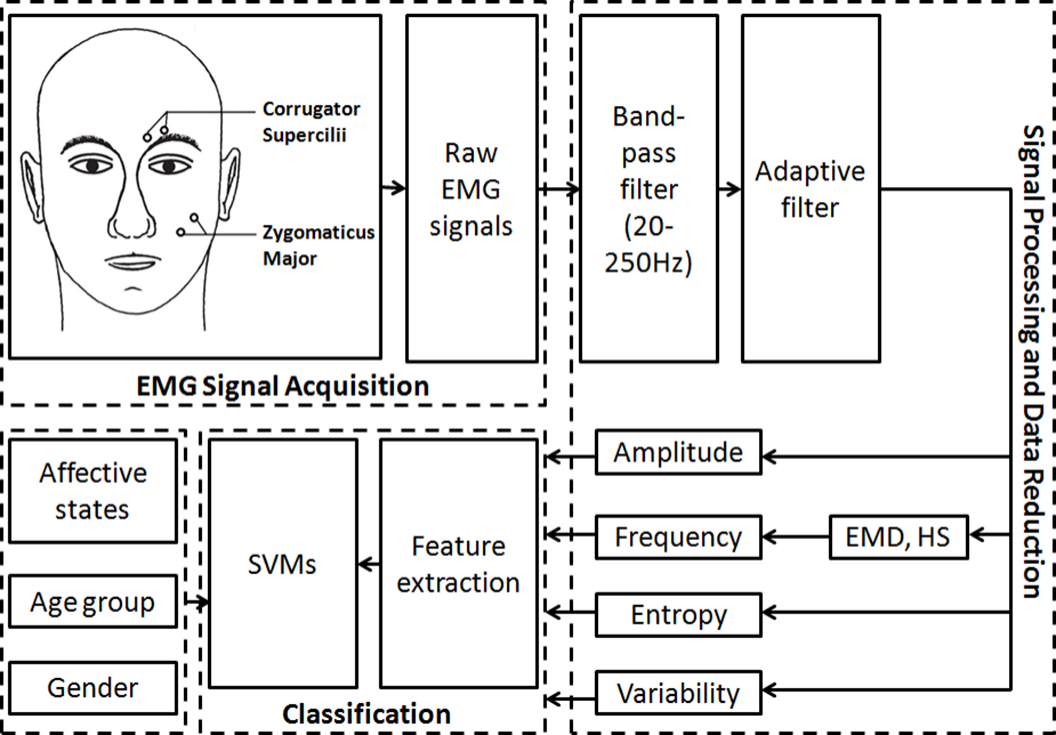
Diagram of EMG signal acquisition, processing, data reduction, and classification.

A NeXus-32 physiological measurement system (*NeXus-32*, *Mind Media*, *Roermond-Herten*, *Netherlands*) running on a desktop computer was used for the acquisition of facial EMG. The software package Biobserve Spectator (*version 2*.*4*.*0*.*5*, *BIOBSERVE GmbH*, *Bonn*, *Germany*) was used to record the trigger and psychophysiological data. Facial EMG signals were captured with bipolar miniature silver/silver chloride (*Ag/AgCl*) skin electrodes 4 mm in diameter with gel-filled attach spaces. Bipolar electrodes were placed on the participants’ left corrugator supercilii and zygomaticus major muscle regions (see Fig 3, EMG signal acquisition) according to the guidelines for EMG placement recommended by Fridlund and Cacioppo [45]. Facial EMG signals were recorded at a sampling rate of 512 Hz.

### EMG Signal Processing and Data Reduction

As shown in Fig 3, raw facial EMG signals were filtered offline by a 20–250 Hz band-pass Butterworth filter (order = 4) to exclude motion-related components, and an adaptive filter was applied to remove the 50 Hz power line interference [46]. In order to classify the stimulated affective statesusing facial EMG, further processing methods including the empirical mode decomposition (EMD) technique and the Hilbert Spectrum (HS) were employed. Data from single (corrugator or zygomaticus) and combined (corrugator and zygomaticus) site(s) were submitted to classification. All the processing, analyses, and machine learning were conducted using the MATLAB software package (version R2009a and R2015a, Mathworks Inc., Natick, MA, USA).

### Data Classification

In order to verify the possibility of discriminating distinct affective states (0VLA, PVHA, NVHA, PVLA, NVLA) and also the baseline (forming a six-class classification problem, in effect), the following sequence of steps for data processing was applied:

1 Feature extraction from particular regions of interest of the signals. A problem we had was defining the length (in seconds) of the region of interest, as there is no specific rule for choosing this length. We assessed distinct empirical combinations as described in Table 1, where similar and distinct periods for baseline and evoked emotions were adopted based on the assumption that evoked emotion could induce early (< 2s) and/or late (> = 2 s) changes in the observed time series.

**Table 1:** Period of time adopted for feature extraction from baseline and evoked emotional periods.

Label	Window length (in seconds) for the baseline period	Window length (in seconds) for evoked emotional period	Description
1–1	1 s	1 s	Features were extracted from the first second for both the baseline and evoked emotional periods
1–2	1 s	2 s	Features were extracted from the first second for the baseline and the first two seconds for evoked emotional periods
1-10-f	1 s	10 s	Features were extracted from the first second for the baseline and the first ten seconds for evoked emotional periods
1-10-l	1 s	10 s	Features were extracted from the first second for the baseline and the last ten seconds for evoked emotional periods
2–2	2 s	2 s	Features were extracted from the first two seconds for both the baseline and evoked emotional periods
2-10-f	2 s	10 s	Features were extracted from the first two seconds for the baseline and the first ten seconds for evoked emotional periods
2-10-1	2 s	10 s	Features were extracted from the first two seconds for the baseline and the last ten seconds for evoked emotional periods

In total, 16 features were estimated from each region of interest, yielding a 16-D feature vector that was properly labeled according to the possible classes (0VLA, PVHA, NVHA, PVLA, NVLA, and baseline). Because two muscles (corrugator and zygomaticus) were examined in this study, the resulting feature vector had 16 (from corrugator or zygomaticus) or 32 (from corrugator and zygomaticus) features. These features were employed according to the literature [47]. The aim of feature extraction was to capture changes in the signals related to their amplitude, frequency, predictability, and variability. Table 2 presents a summary of the employed features, and a comprehensive description of the features is provided in a previously published study [48].

**Table 2:** Feature descriptions.

Feature group	Feature	Description
Amplitude	Mav	mav = mav(signal)
	Mavfd	mavfd = mavfd(signal)
	Mavsd	mavsd = mavsd(signal)
	Peak	peak = max(signal); index(max(signal))
	Rms	rms = rms(signal)
Frequency	Zc	Calculated by comparing each point of the signal with the next; if there is a crossing by zero then it is accounted.
	Fmed	To obtain the median frequency, find the value of the frequency that bisects the area below the X waveform.
	Fmode	This fast Fourier transformation equation is valid for this and the following frequency features:. $X\left(k\right)=\sum_{j=1}^{N}x\left(j\right)\omega_{N}{}^{\left(j-1\right)\left(k-1\right)}$, where $\omega_{N}=e^{\left(-\frac{2\pi i}{N}\right)}$. To find the mode, find the maximum value of X.
	Fmean	$\frac{\sum_{k=1}^{NFFT}X\left(k\right).f\left(k\right)}{\sum X\left(k\right)}$
	Cf	The central frequency is simply the mean of the frequencies that delimit the bandwidth: $cf=\frac{fh-fl}{2}$.
Predictability	Fuzzy entropy	$Saen\left(m,s,d\right)=\ln\left[\frac{Co_{m}\left(s\right)}{Co_{m+1}\left(s\right)}\right]$, where *m* is the window size, *s* is the similarity standard and *d* is the signal. It is calculated in a very similar way to the Sample Entropy. The only similarity between the groups is computed by means of a Fuzzy membership function.
	Approximate entropy	For a temporal series with N samples {*u*(*i*): 1 ≤ *i* ≤ *N*} given *m*, create vectors $X_{j}^{m}$ for each $X_{N-\text{m}+1}^{m}$ as $X_{j}^{m}=\left\{u\left(i\right),u\left(i+1\right),u\left(i+m-1\right)\right\},i=1,\ldots ,N-m+1$, where *m* is the number of points to group together for the comparison. For each *k* ≤ *N* − *m* + 1 groups, do $C_{k}^{m}\left(r\right)$ which is the number of times the groups had distance less than tolerance *r*. Then compute the value *φ*^*m*^ as $\varphi^{m}\left(r\right)=\frac{\sum_{i=1}^{N-m+1}\ln C_{j}^{m}\left(r\right)}{N-m+1}$. The Approximated Entropy is: *ApEn*(*m*,*r*) = lim_*N*→∞_[*φ*^*m*^(*r*) − *φ*^*m*+1^(*r*)].
Variability	Var	$\sigma^{2}=\frac{\sum_{i=1}^{N}{\left(x^{i}-\overset{-}{x}\right)}^{2}}{N-1}$
	Std	$S=\sqrt{\sigma^{2}}$
	Range	*R* = *MAX*(*U*) − *MIN*(*U*)
	Intrange	$SI=\frac{Q3-Q1}{2}$

2 Features extracted from all subjects were organized in a table in which each column represented a feature or variable. Prior to classification, each feature was standardized to have zero mean and unit variance. Classification was performed with the machine learning toolbox available in Matlab 2015Ra (Mathworks). The support vector machine (SVM) classifier was used with a Gaussian kernel (i.e, *templateSVM*['KernelFunction','gaussian'] in the Matlab environment.) The *fitcecoc* method available in Matlab, which fits multiclass models for support vector machines or other classifiers, was employed, and 10-fold cross-validation was used for classifier assessment.
3 We estimated the success rate of each class, defined as the number of correctly classified patterns divided by the total number patterns of the class. The entire classifier-training procedure and performance assessment was repeated 10 times to estimate the mean and the standard deviation of the rate of success.

## Results

### Classification Results

The classification results are presented from Tables 3–7, where results are presented for each specific condition listed in Table 1.

**Table 3:** M and SD of classification accuracy across baseline and five affective states via the combination of corrugator and zygomaticus EMG data for all participants.

		Baseline (%)	0VLA (%)	PVHA (%)	NVHA (%)	PVLA (%)	NVLA (%)
1–1	M	100.00	88.47	88.38	87.31	88.47	87.04
	SD	0.00	0.60	0.63	0.54	0.41	0.49
1-10-f	M	100.00	88.75	86.02	84.95	84.81	86.90
	SD	0.00	0.73	0.87	0.82	0.81	0.90
1-10-l	M	100.00	88.75	86.20	84.58	85.28	87.36
	SD	0.00	0.82	0.65	1.18	1.11	0.76
1–2	M	100.00	90.65	89.44	87.92	89.72	89.95
	SD	0.00	0.48	0.65	0.63	0.68	0.62
2–2	M	100.00	75.69	77.13	80.69	79.72	76.57
	SD	0.00	1.47	0.98	0.58	0.89	1.00
2-10-f	M	100.00	89.07	85.88	85.19	85.46	87.27
	SD	0.00	1.03	1.05	0.87	1.10	0.91
2-10-l	M	100.00	88.47	86.81	85.05	85.37	87.45
	SD	0.00	0.91	0.82	0.66	0.73	1.49

**Table 4:** M and SD of classification accuracy across baseline and five affective states via the combination of corrugator and zygomaticus EMG data for young and senior participants.

			Baseline (%)	0VLA (%)	PVHA (%)	NVHA (%)	PVLA (%)	NVLA (%)
1–1	Young	M	100.00	88.60	87.87	88.82	88.01	87.13
		SD	0.00	0.39	0.79	0.47	0.70	0.62
	Senior	M	100.00	90.00	90.00	90.00	90.00	90.00
		SD	0.00	0.00	0.00	0.00	0.00	0.00
1-10-f	Young	M	100.00	88.75	85.96	83.82	84.78	85.96
		SD	0.00	1.73	1.32	0.78	1.04	1.45
	Senior	M	100.00	89.63	89.75	88.50	90.13	89.88
		SD	0.00	0.84	0.53	0.79	0.40	0.71
1-10-l	Young	M	100.00	88.75	86.69	84.41	85.96	85.81
		SD	0.00	0.98	1.17	1.54	1.68	1.63
	Senior	M	100.00	90.25	89.88	89.00	90.00	90.13
		SD	0.00	0.79	0.40	0.79	0.83	0.71
1–2	Young	M	100.00	90.74	89.19	88.46	89.63	90.00
		SD	0.00	0.71	0.36	0.36	0.54	0.38
	Senior	M	100.00	90.00	90.00	90.13	90.00	90.00
		SD	0.00	0.00	0.00	0.40	0.00	0.00
2-10-f	Young	M	100.00	89.04	86.62	84.26	86.32	86.47
		SD	0.00	0.64	1.38	0.86	0.86	1.05
	Senior	M	100.00	90.00	89.50	89.13	89.88	89.88
		SD	0.00	0.59	0.65	0.60	0.71	1.38
2-10-l	Young	M	100.00	88.97	86.69	83.75	85.22	86.10
		SD	0.00	0.60	1.36	1.12	1.36	1.32
	Senior	M	100.00	90.13	89.38	88.75	89.38	90.00
		SD	0.00	0.92	0.88	0.00	1.35	1.02
2–2	Young	M	99.99	77.65	78.31	81.76	77.06	78.46
		SD	0.05	1.21	1.05	0.90	1.62	1.55
	Senior	M	100.00	89.00	88.63	87.88	88.88	86.25
		SD	0.00	1.15	0.71	0.84	0.92	1.02

**Table 5:** M and SD of classification accuracy across baseline and five affective states via the combination of corrugator and zygomaticus EMG data for female and male participants.

			Baseline (%)	0VLA (%)	PVHA (%)	NVHA (%)	PVLA (%)	NVLA (%)
1–1	Female	M	100.00	90.07	89.56	89.49	89.41	89.49
		SD	0.00	0.39	0.58	0.36	0.38	0.36
	Male	M	100.00	88.88	90.00	90.00	90.00	88.75
		SD	0.00	0.40	0.00	0.00	0.00	0.00
1-10-f	Female	M	100.00	87.65	87.06	86.25	87.28	86.62
		SD	0.00	1.24	1.16	1.20	0.92	1.19
	Male	M	100.00	89.38	88.00	89.88	89.38	90.00
		SD	0.00	1.06	1.05	0.71	0.66	0.59
1-10-l	Female	M	100.00	86.76	87.94	86.40	87.06	86.91
		SD	0.00	1.73	1.21	1.05	1.21	1.09
	Male	M	100.00	89.00	88.00	90.13	89.38	90.25
		SD	0.00	0.79	0.65	0.40	1.06	1.29
1–2	Female	M	100.00	90.59	90.59	90.00	89.71	89.85
		SD	0.00	0.31	0.47	0.62	0.00	0.31
	Male	M	100.00	90.00	90.00	90.38	89.88	89.00
		SD	0.00	0.00	0.00	0.60	0.40	0.53
2-10-f	Female	M	100.00	86.25	87.94	85.44	87.43	86.47
		SD	0.00	1.20	0.93	1.38	1.12	1.11
	Male	M	100.00	88.88	88.38	90.13	89.50	90.00
		SD	0.00	0.71	1.03	0.92	0.87	0.83
2-10-l	Female	M	100.00	87.43	87.50	85.81	87.43	86.91
		SD	0.00	0.88	1.20	1.04	1.07	1.62
	Male	M	100.00	89.13	88.75	90.00	89.38	89.88
		SD	0.00	1.03	0.83	0.59	0.66	1.09
2–2	Female	M	99.97	81.18	84.04	81.32	82.57	79.41
		SD	0.06	0.71	1.04	1.11	1.15	0.98
	Male	M	100.00	87.63	87.25	85.00	85.50	87.38
		SD	0.00	0.40	0.99	1.32	1.05	0.92

**Table 6:** M and SD of classification accuracy across baseline and five affective states via solely corrugator EMG data for all participants.

		Baseline (%)	0VLA (%)	PVHA (%)	NVHA (%)	PVLA (%)	NVLA (%)
1–1	M	99.87	22.22	34.07	38.94	34.68	30.83
	SD	0.10	1.43	1.97	1.84	1.60	2.27
1-10-f	M	100.00	55.23	61.20	56.34	60.19	62.87
	SD	0.00	2.58	3.04	2.70	2.43	2.50
1-10-l	M	100.00	56.53	62.18	56.81	60.14	61.85
	SD	0.00	3.58	1.50	2.40	1.41	2.78
1–2	M	100.00	68.10	69.86	65.42	63.89	65.88
	SD	0.00	2.74	1.55	1.73	2.82	3.18
2-10-f	M	100.00	55.83	61.67	56.71	59.12	61.90
	SD	0.00	3.17	2.44	2.19	3.13	3.19
2-10-;	M	100.00	55.60	61.25	56.57	58.80	61.94
	SD	0.00	3.64	2.91	2.33	2.56	3.06
2–2	M	99.91	14.03	23.38	34.31	25.09	25.46
	SD	0.04	1.00	1.24	1.71	1.23	1.84

**Table 7:** M and SD of classification accuracy across baseline and five affective states via solely zygomaticus EMG data for all participants.

		Baseline (%)	0VLA (%)	PVHA (%)	NVHA (%)	PVLA (%)	NVLA (%)
1–1	M	99.95	19.68	17.55	19.31	20.65	19.17
	SD	0.09	0.88	1.41	1.38	1.59	1.35
1-10-f	M	100.00	54.81	52.69	55.93	52.50	61.67
	SD	0.00	3.63	1.59	3.24	1.35	4.05
1-10-l	M	100.00	54.58	52.96	56.44	50.65	62.92
	SD	0.00	1.83	2.12	3.21	1.79	3.43
1–2	M	100.00	60.00	54.81	57.36	54.81	65.93
	SD	0.00	2.18	1.97	3.14	2.12	2.50
2-10-f	M	100.00	54.40	52.87	56.34	51.20	63.01
	SD	0.00	3.10	1.41	2.13	1.29	3.00
2-10-l	M	100.00	54.44	52.45	55.79	51.30	64.58
	SD	0.00	1.59	0.90	3.08	1.32	3.64
2–2	M	99.94	13.19	18.24	20.93	17.59	18.61
	SD	0.09	2.35	1.18	0.72	1.43	1.56

Table 3 describes the mean (M) and standard deviation (SD) of classification accuracies (ten-fold cross-validation) of five affective states and the baseline, based on the combined corrugator and zygomaticus EMG data. The classification accuracy reached 100% for the baseline in all seven conditions, where the five affective states ranged from 87.04% to 88.47% in 1–1, from 87.92% to 90.65% in 1–2, from 84.81% to 88.75% in 1-10-f, from 84.58% to 88.75% in 1-10-l, from 75.69% to 80.69% in 2–2, from 85.19% to 89.07% in 2-10-f, and from 85.05% to 88.47% in 2-10-l.

Table 4 presents the M and SD classification accuracies of baseline and the five affective states based on the combined corrugator and zygomaticus EMG data according to age. For the baseline, the classification accuracy hit 100% for all conditions except in 2–2 in young groups; the five affective states ranged from 77.06% to 90.74% in the young group and from 86.25% to 90.25% in the senior group. The classification rates for senior adults were higher than young adults in all seven conditions.

As far as gender, as Table 5 shows, classifying the combination of corrugator and zygomaticus EMG showed similar results regardless of age; the baseline was nearly 100% in all conditions except in 2-2in the female group. The five affective states ranged from 81.18% to 90.59% in the female group and from 85.00% to 90.38% in the male group.

Tables 6 and 7 show where the M and SD of classification accuracy for the baseline reached nearly 100% with either single corrugator or zygomaticus EMG; all five affective states are relatively low (from 13.19% to 69.86%).

### Statistical Results

To compare the effects of single corrugator and zygomaticus and the combination of corrugator and zygomaticus EMG for affect recognition, data from Tables 3, 6 and 7 were submitted to repeated measures ANOVA analysis. The results demonstrated a significant primary effect (*F*(1,41) = 87.34, *p*< 0.001), and multiple comparisons with Bonferroni revealed significant differences between the combination of corrugator and zygomaticus and the single corrugator, the combination of corrugator and zygomaticus and single zygomaticus, and the single corrugator and zygomaticus (*p* < 0.001).

As far as age, data from Table 4 was subjected to the independent T-test, and results showed significant differences between young and senior participants (*t* = -2.67, *p* < 0.01, two-tailed).

Data from Table 5 was subjected to independent T-test, with no pronounced difference between female and male subjects (*t* = -1.58, *p* > 0.05, two-tailed).

## Discussion and Conclusions

The interaction between humans and machines will, in future, most likely be an empathic relationship resembling the “companionship” observed in HMI research. In such a companionship, the ability to perceive a user’s current physical conditions and emotional state is highly desirable [1]. In other words, the ideal companion system is able to fully empathize with its individual user [3–5]. To achieve this, artificial “eyes” and “ears” such as cameras, microphones, and sensors are required. These physical devices, including psychobiological sensors, provide accurate signals that allow the machine to gain access to human emotional states.

The primary goal of this study was to investigate the performance of facial EMG for recognizing valence-arousal affective states, in effort to bridge the gap between machine and human emotional experiences. We specifically focused on the classification of intensive valence and arousal affective states on facial EMG activities captured over corrugator supercilii and zygomaticus major, as well as age and gender differences.

Future, successful application of facial EMG for identifying affective states in HMI must be in real-time–the machine-learning system could offer us the opportunity to classify affective states automatically. With the combination of corrugator and zygomaticus EMG data, the classification rate achieved high accuracies from 75.69–100.00% for the baseline, 0VLA, PVHA, PVLA, NVHA, and NVLA in all individuals, young and senior groups, female and male participants. In contrast, by using single corrugator or zygomaticus EMG data, the classification rate for the baseline reached a perfect level while the other five affective states reached only a very low level. Basically, combining a separated corrugator as well as azygomaticus EMG would be the better way for affect classification.

One interesting finding of this study is that the classification accuracy for senior adults was significantly higher than that for young adults. To our knowledge, there has been no consensus achieved for age differences on emotion recognition, especially for combined intensive valence and arousal states. As such, our findings provide valuable new information on the topic.

No significant difference due to gender was found in the classification results, which confirms published findings that though women may recognize emotion more accurately, they show no difference in intensive affective states compared to men [48].

One limitation of this study is that it only employed facial EMG; other psychobiological channels such as skin conductance level (SCL), supposedly associated with arousal, could help to differentiate valence and arousal affective states. Future research should consider these shortcomings and increase the sample size. Moreover, similar to the interpersonal interaction situation, in HMI, users’ expectations regarding cooperation and competition affect their emotional responses [49]. Future studies investigating possible confounds of expectation, motivation, personality, gender, and neurological or psychiatric conditions are necessary due to the complexity of individuals and machine companionship goals.

The emotion dimension, in which valence emotions have received much attention using facial EMG, is predominantly employed. One dimension is not enough, though, so this study went beyond the single dimension of valence. To the best of our knowledge, no study has investigated the effect of dominance on facial EMG, even though dominance is assumed to strongly correlate with valence. This should also be considered in future studies of emotion recognition through facial EMG.

In conclusion, the facial EMG technique for differentiating valence-arousal emotions was indeed confirmed by the results of our experiments. Similar to conditions in natural circumstances, in HMI, emotional experiences consist of many elementary emotions that may rapidly change. Thus, facial EMG response patterns may indicate dynamic emotional states [50]. Moreover, the procedure employed to measure emotions induced by a standardized set of affective visual stimuli in this study may contribute to methods for successfully identifying individual situations in HMI. Future applications, such as real-time calibration methods for emotion recognition in machines or even in companion robotic systems and for users with personalized needs in healthcare settings, may be informed by the results of this study [51].

## Supporting Information

S1 Data Data of features for 7 conditions. In this dataset, there are 7 excel files. Each of them includes 32 features (from corrugator and zygomaticus EMG) for certain combination of different period of baseline and evoked emotions.
(RAR)

S2 Data Data of classification results. In this dataset, there are 3 excel spss files: Classification Results vs. Age, Classification Results vs. Gender, Classification Results vs. Corrugator, Zygomaticus, Corrugator and Zygomaticus.
(RAR)
